# Supplementary material for: A Novel Curcumin-Mycophenolic Acid Conjugate Inhibited Hyperproliferation of Tumor Necrosis Factor-Alpha-Induced Human Keratinocyte Cells
Source: Pharmaceutics. 2021 Jun 25;13(7):956. doi: 10.3390/pharmaceutics13070956 (PMC8308932; doi:10.3390/pharmaceutics13070956)
Supplement: Supplementary file 1 [file pharmaceutics-13-00956-s001.zip › pharmaceutics-1244303-SI.pdf]

pharmaceutics

Supplementary Materials: A Novel Curcumin-Mycophenolic Acid Conjugate Inhibited Hyperproliferation of Tumor Necrosis Factor-Alpha-Induced Human Keratinocyte Cells

Yonelian Yuyun, Pahweenvaj Ratnatilaka Na Bhuket, Wiwat Supasena, Piyapan Suwattananuruk, Kemika Praengam, Opa Vajragupta, Chawanphat Muangnoi and Pornchai Rojsitthisak

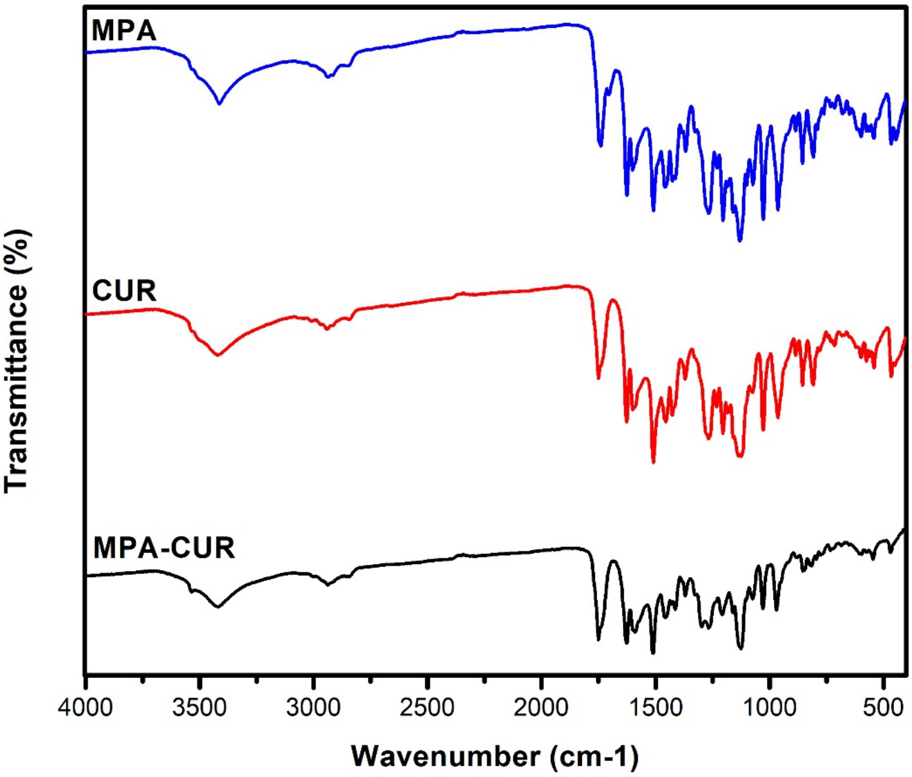

Figure S1. IR spectrum of MPA, CUR, and MPA-CUR conjugate.

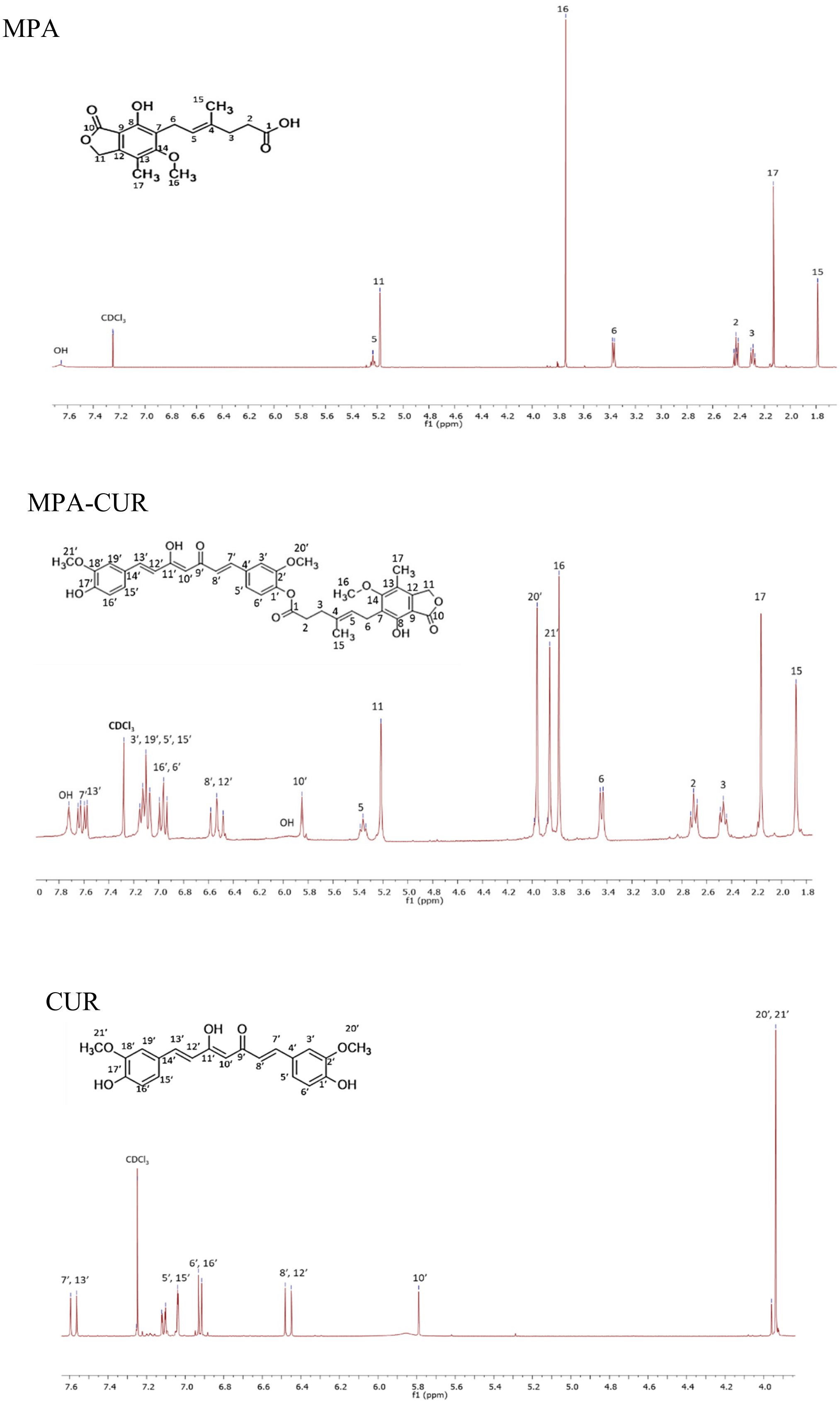

Figure S2. ^{1}H-NMR spectrum of MPA, CUR and MPA-CUR conjugate.

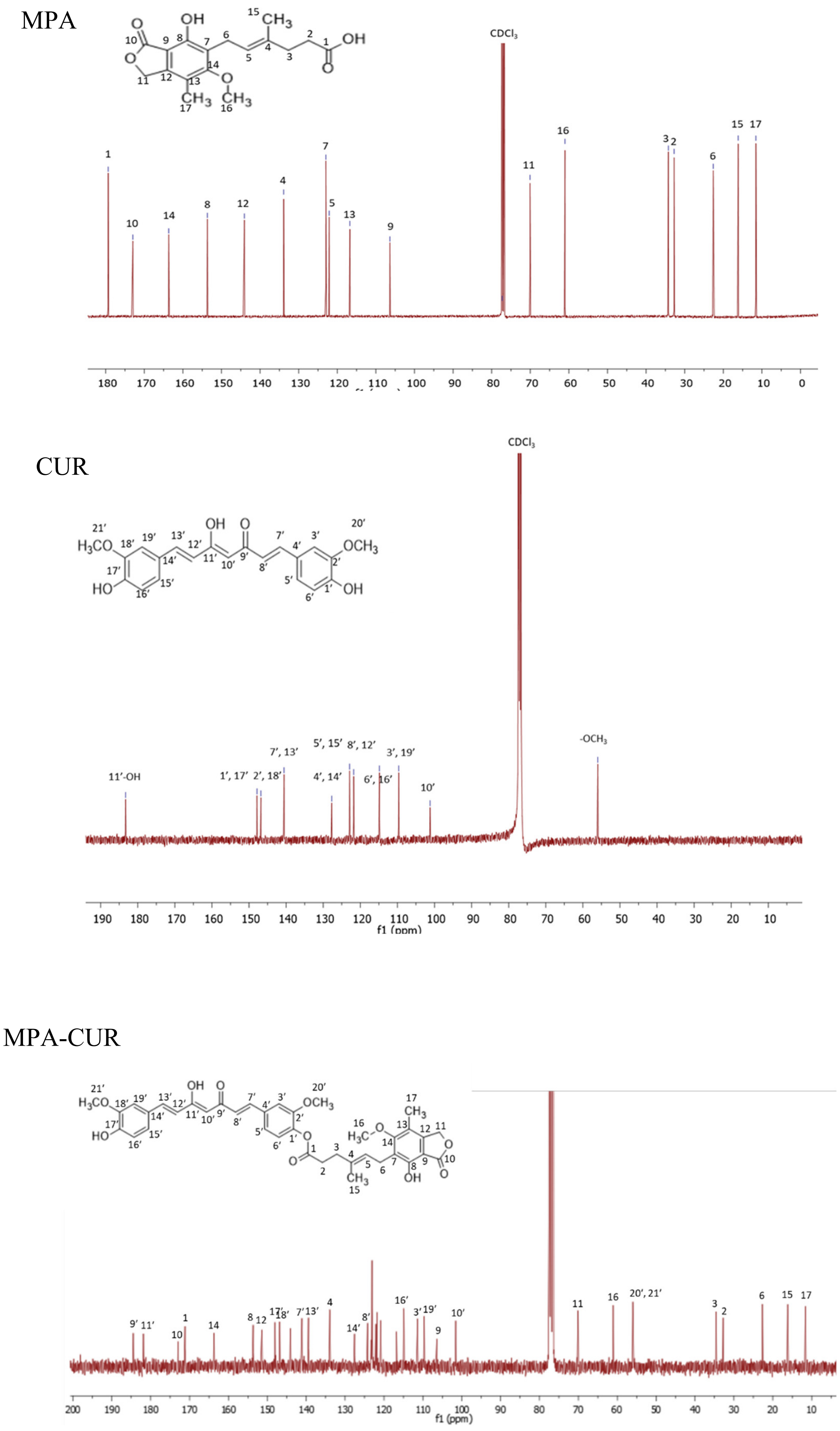

Figure S3. ^{13}C-NMR spectrum of MPA, CUR and MPA-CUR conjugate.

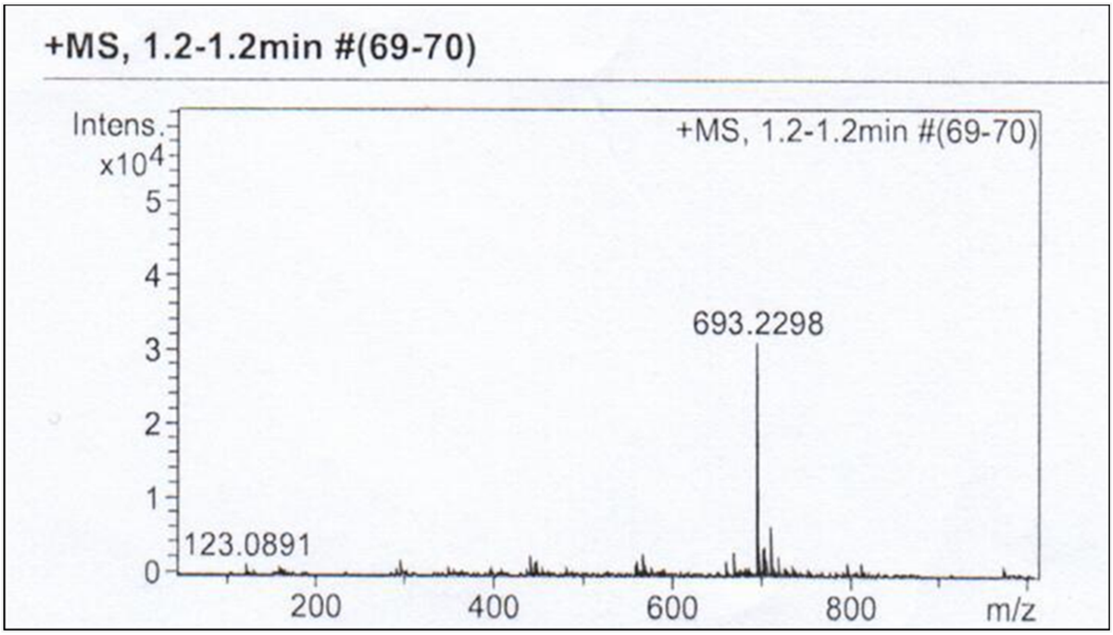

Figure S4. MS spectrum of MPA-CUR.
